# Case report: A novel mosaic nonsense mutation of *PCDH19* in a Chinese male with febrile epilepsy

**DOI:** 10.3389/fneur.2022.992781

**Published:** 2022-09-29

**Authors:** Guilan Chen, Hang Zhou, Yan Lu, You Wang, Yingsi Li, Jiaxin Xue, Ken Cheng, Ruibin Huang, Jin Han

**Affiliations:** ^1^Department of Prenatal Diagnostic Center, Guangzhou Women and Children's Medical Center, Guangzhou Medical University, Guangzhou, China; ^2^The First Clinical Medical College, Southern Medical University, Guangzhou, China; ^3^School of Medicine, South China University of Technology, Guangzhou, China

**Keywords:** *PCDH19*, febrile seizures, mosaicism, case report, exome sequence

## Abstract

The clinical features of the *PCDH19* gene mutation include febrile epilepsy ranging from mild to severe, with or without intellectual disability, cognitive impairment, and psych-behavioral disorders, but there has been little research on males with the mosaic mutation of *PCDH19*. This study reported a novel, *de novo*, and mosaic *PCDH19* nonsense mutation (NM_001184880: c.840C > A, p. Tyr280^*^) from a Chinese male in early middle childhood by trio whole-exome sequence (Trio-WES) and confirmed by Sanger sequence. The proportion of the mosaic mutation (c.840C > A, p. Tyr280^*^) in *PCDH19* was 27.9% in, buccal mucosal cells, 48.3% in exfoliated cells in the urine, and 50.6% in peripheral blood of proband. He had the first onset of seizures in toddlerhood with febrile epilepsy, mild impaired cognitive psychological, and behavioral abnormalities. The electroencephalography (EEG) exhibited sharp waves and sharp slow complex waves in the bilateral parietal, occipital, and posterior temporal regions during the interictal period. Pinpoint white matter lesions in the periventricular white matter and slightly bulging bilateral ventricles appeared on cranial magnetic resonance imaging (MRI). With Depakine and Keppra he gained good control over his epilepsy. This study might expand the genotypes and broaden the spectrums.

## Introduction

With the wide application of next-generation sequence (NGS) technology, the etiology of epilepsy is gradually associated with multiple genes. Protocadherin 19 (*PCDH19*) is one of the most common genetic causes of epilepsy (1). Interestingly, *PCDH19*-caused X-linked developmental and epileptic encephalopathy 9 (OMIM: 300088), characterized by unusual epilepsy with or without fever sensitivity, with or without cognitive impairment, and psychoneurological disorder, is common to most females with heterozygous variation and males restricted with mosaic mutation, but not hemizygous males (2). The pathogenesis of this unique disorder could be partially explained by a cellular interference mechanism between mutant-type neural cells and normal neural cells in the developing brain (3). Several studies in the *PCDH19* show that the severity of encephalopathy is related to the onset time of epilepsy, the proportion of mosaicism, and the types of mutation (such as missense and truncating mutations) and the domains affected by mutations (4–7). To date, more than 270 pathogenic variants have been reported in *PCDH19* according to Human Gene Mutation Database (HGMD). However, most research has focused on the affected females with the *PCDH19*-related disorder, but males with pathogenic *PCDH19* variation have rarely been reported. Here, we report a novel mosaic *PCDH19* nonsense mutation (NM_001184880: c.840C > A, p. Tyr280^*^) in a Chinese 6 year old boy with febrile epilepsy and mild impaired cognitive psychological, and behavioral abnormalities. He gained long-term control (>12 months) of his epilepsy on anti-epileptic drugs, including Depakine and Keppra. This study might expand the genotypes and broaden the spectrums.

## Case presentation

**Figure 1:**
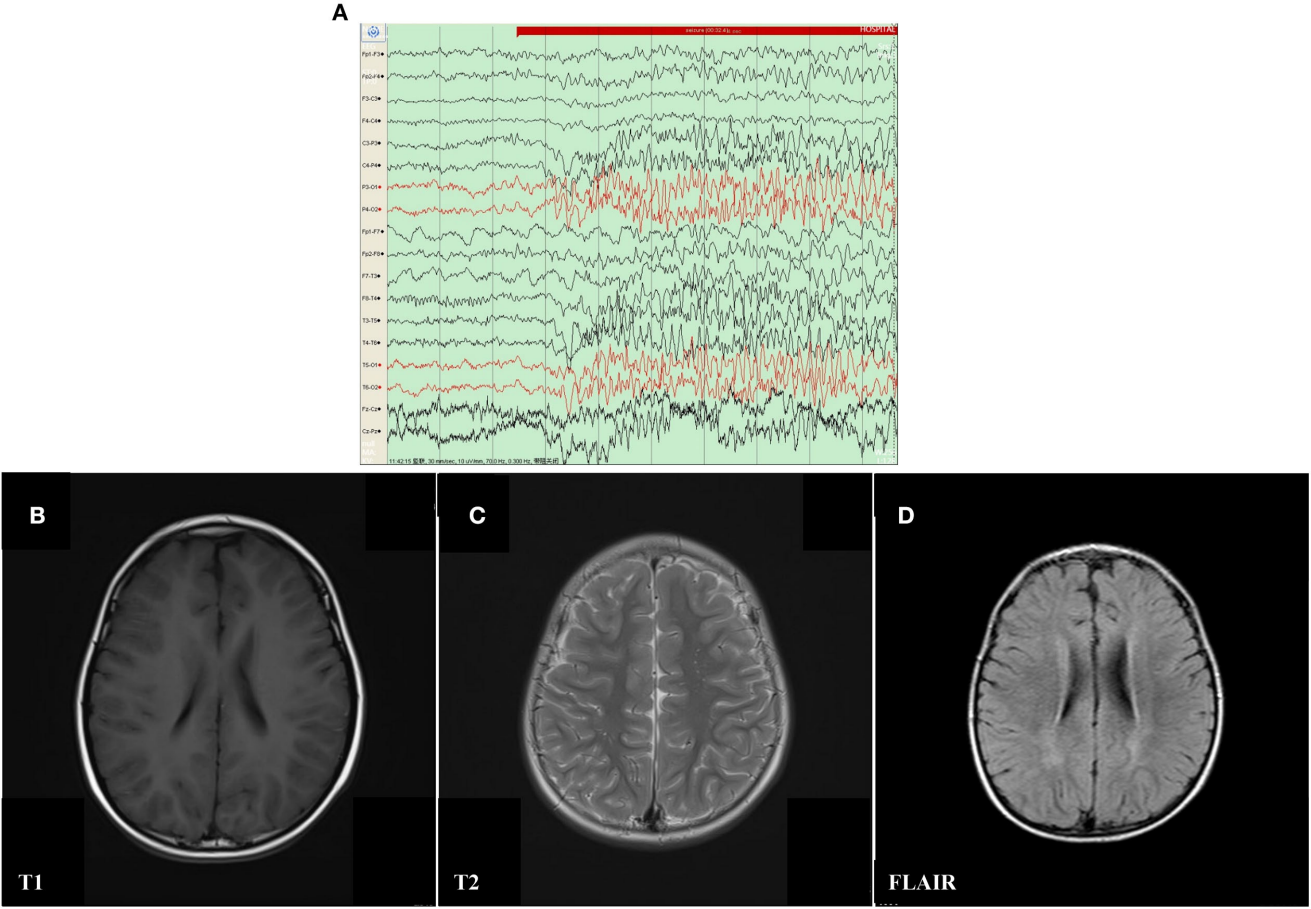
Electroencephalography (EEG) and Cranial magnetic resonance imaging (MRI) result of proband with mosaic mutation in PCDH19 gene (c.840C>A, p. Tyr280*). **(A)** The analysis of electroencephalography (EEG) exhibited that sharp waves and sharp slow complex waves in the bilateral parietal, occipital, and posterior temporal regions were distributed locally with normal background during the interictal period. **(B–D)** Punctate white matter lesions appeared with slightly long T1 and T2 signals, as well as slightly high signal on FLAIR in the periventricular white matter and slightly plump bilateral ventricles.

The proband was a 6 year old boy. He was the first child of his parents who were both healthy and not consanguineous. His birth history and perinatal history were not special. He was born by spontaneous delivery with a 3.1 kg birth weight. There was no positive family history of epilepsy. The febrile generalized tonic-clonic seizures were present in his 14 month old triggered by mycoplasmal pneumonia for the first time. The seizures lasted approximately 2 min and remitted spontaneously, with seizures occurring 5 times in 15 h. The analysis of electroencephalography (EEG) exhibited that sharp waves and sharp slow complex waves in the bilateral parietal, occipital, and posterior temporal regions were distributed locally with normal background during the interictal period (see Figure 1A). By cranial magnetic resonance imaging (MRI), punctate white matter lesions appeared with slightly long T1 and T2 signals, and slightly high signals on fluid-attenuated inversion recovery (FLAIR) in the periventricular white matter and slightly plump bilateral ventricles (see Figures 1B–D). Mycoplasma pneumonia antibodies IgM was positive with high titers. The other examinations were not found abnormal including the routine blood test, coagulative function evaluation, liver, and renal function, autoimmune antibodies, blood ammonia, plasma amino acids, plasma acylcarnitine, urine analysis of gas chromatography-mass spectrometry (GC–MS), routine examination, and biochemical detection of cerebrospinal fluid (CSF) acquired by lumbar puncture. Phenobarbital, midazolam, and Keppra were initially used for the boy, but epilepsy remained poorly controlled by this treatment. After Depakine and methylprednisolone were subsequently added, his seizure was gradually controlled.

During hospitalization for the first seizure, in the absence of a molecular diagnosis, the boy was diagnosed with viral encephalitis epilepsy by a neurologist. Therefore, after discharge, he continued to receive methylprednisolone to improve and reduce inflammation in the central nervous system. In addition, he insisted on using sodium valproate, levetiracetam (LEV), and oxiracetam to control and prevent seizures. Seizures were not observed for approximately 3 years and 10 months.

After a 42 month seizure-free period, at the age of 5 years and 2 months, he developed a second generalized tonic-atonic febrile seizure after a fall, which lasted about 5–10 min and resolved spontaneously. The seizures recurred after 1 h and lasted about 10 min. Seizures were well controlled by oral Depakine, Keppra, and other supportive care after admission. Epileptic seizures were not observed in him for 21 months till the last follow-up. Similar to previous results, the EEG revealed local sharp waves and sharp slow waves in the right parietal and middle temporal areas and the MRI was identical to the previous test the first time. There were no abnormal findings in other examinations, such as quantitative detection of EB virus DNA, cytomegalovirus DNA, enterovirus and herpes simplex virus, auditory evoked potential, visual evoked potential, and transcranial Doppler ultrasound (TCD).

Developmentally, he could raise his head at 4 months, sit and roll at 7 months, and walk at 12 months. After suffering from first febrile seizures, he had remarkable developmental delay and progressive motor disturbances. Subsequently, the onset of seizures for the second time, he could not walk or talk within a week. The WPPSI-IV (Wechsler Preschool and Primary Scale of Intelligence) showed that his full-scale intellectual quotient (FSIQ) was 79 at his age of five. He had a verbal comprehension index (VCI) of 79 and a normal evaluation of social-life ability.

**Figure 2:**
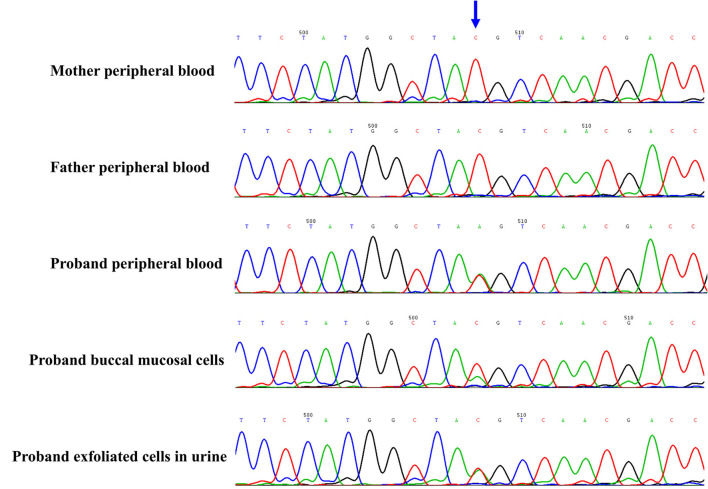
The novel de novo mosaic mutation in PCDH19 gene in proband confirmed by Sanger sequence. The variant was identified in proband but absent in parents. The proportion of mosaic mutation (c.840C>A, p. Tyr280*) in PCDH19 gene was 27.9% in, buccal mucosal cells, 48.3% in exfoliated cells in urine and 42.4% in peripheral blood of proband.

The proband and his parents received a trio-WES (whole-exome sequence) and copy number variants (CNVs) analysis by detecting DNA extracted from peripheral blood. The CNVs analysis was negative. However, trio-WES identified a novel pathogenic mosaic nonsense mutation of the *PCDH19*, NM_001184880: c.840C > A, p. Tyr280^*^, affecting the exon 1 involved extracellular domain of the *PCDH19*. This mutation was predicted to be pathogenic (PVS1 + PS2 + PM2). The heterozygous variant was confirmed by the Sanger sequence (see Figure 2). This variant was *de novo* and absent in his parents. The proportion of mosaicism was 53 and 50.6% according to WES and Sanger sequence results, respectively. Interestingly, the proportion of mosaic mutation (c.840C > A, p. Tyr280^*^) in *PCDH19* was 27.9% in, buccal mucosal cells and 48.3% in exfoliated cells in urine, respectively.

## Materials and methods

This study was approved by the ethics committee of Guangzhou Women and Children's Medical Center. The trio WES (whole exome sequence) was conducted with 200X average coverage after informed consent. The patient was diagnosed with epilepsy by reference to the International League Against Epilepsy definition. Clinical information of the patient was recorded including medical history, family history, mental growth and development, electroencephalography (EEG), magnetic resonance imaging (MRI), hospitalization data and drug use et. al. The pathogenicity of the variant was classified according to standards and guidelines of the American College of Medical Genetics and Genomics. DNA libraries were prepared using a NEXTflex^TM^ Rapid DNA Sequencing Kit (5144-02) according to the manufacturer's protocol. Exome sequencing was enriched in the DNA sample by using Agilent SureSelect human exome capture probes (V6, Life Technologies, USA) according to the manufacturer's protocol. The whole exome sequence was performed by utilizing Hiseq XTen (Illumina, Inc., San Diego, CA, USA) for pair-end 150-bp reads. Trimmomatic was used to remove adapter contaminated reads and low-quality for raw reads filtering. Clean reads were aligned to the human reference genome (NCBI GRCh37/hg19) with the BWA mem algorithm. BAM files were conducted for SNP analysis, duplication marking, indel realignment, and recalibration using SAMtools and GATK. The minor allele frequencies (MAFs) of all known variants were annotated according to dbSNP, the 1,000 Genome Project, ExAC, EVS, gnomAD, and our in-house database. Databases such as OMIM, ClinVar, and Human Gene Mutation Database were used to determine mutation harmfulness and pathogenicity where appropriate. The biological effects analysis of candidate variant genes was determined by using programs SIFT, MutationTaster, PolyPhen2, PROVEAN, CADD, Human Splicing Finder, MaxEntScan, and NNSplice. The variants suspected to be of clinical significance were confirmed by Sanger sequencing methods. The ratios of mosaic mutation in the peripheral blood, exfoliated cells in the urine, and buccal mucosal cells were calculated by Bioedit software by analyzing the Sanger sequencing results.

## Discussion

*PCDH19*-associated epilepsy was the second most common cause of epilepsy. To date, there have been over 270 variants of *PCDH19* in females. The phenotypes were considered in previous studies to be restricted to women with the *PCDH19* heterozygous mutation. However, there have been few reports of male epileptic patients with mosaic *PCDH19* mutations. This study reported a mosaic, *de novo*, and nonsense mutation in exon 1 of *PCDH19* (c.840C > A, p.Tyr280^*^) in a 6 year old boy with febrile epilepsy.

Although there was no genotype and phenotype correlation from a 271 meta-analysis study in 2018 (4), Shibata et al. (5) found truncating variants from extracellular domain 5 (EC5) to cytoplasmic domain showed later seizures and less intellectual disability, compared with missense variants and truncating variants from EC1 to EC4. The *PCDH19* belongs to the δ2-protocadherin subclass of the cadherin superfamily and is located on the X chromosome (Xq22.1), which encodes a total of six exons (8). The first exon encodes the extracellular, transmembrane domains, and a small portion of the C-terminal region. The left C-terminal region is coded by exon 2–6 (9). Exon 1 is unusually large and long, as well as contains the most numbers of pathogenic variants among all exons in *PCDH19* (4, 10). Furthermore, there were more pathogenic variants per base (5% per base) in exon 1 of the *PCDH19* (4). It suggested that variation in extracellular regions was poorly tolerated. The position of the nonsense mutation in our case was at exon 1 of the *PCDH19* (c.840C > A, p. Tyr280^*^). The same location of the mosaic *de novo* variant (c.840C > G, p. Tyr280^*^) has been previously reported in a male (6). Interestingly, there were many parallels in phenotypes between the report and our case. The case with a similar variation in *PCDH19* had generalized clonic epilepsy accompanied by fever sensitivity at the tenth month after birth. He also had a cognitive disorder, psychological, and behavioral abnormalities. A previous study suggested that there were milder phenotypes in cases with a missense mutation in the extracellular cadherin (EC) domain 5 (5). But in our case, theoretically, the truncation variant of *PCDH19* should have resulted in a severe phenotype, but in this case there is mild epilepsy, no developmental delay, and psychiatric disorders. We speculated that this is more related to the late age of onset and the low proportion of oral mosaic mutations, which might reflect the proportion of abnormal neurons. Further studies are needed such as how the *PCDH19* mutations are involved in febrile epilepsy, what the differences in pathogenesis are among various types of variation and what the factors are that lead to incomplete penetrance.

The first onset time of seizures was negatively correlated with the poor prognosis of epilepsy. In this study, our case, who developed epilepsy for the first time at 14 months postpartum, presented with mild cognitive impairment and psychological and behavioral abnormalities. In addition, he gained long-term control (>12 months) of epilepsy on anti-epileptic drugs, including Depakin and Keppra. A meta-analysis including 271 cases with the *PCDH19* variants demonstrated seizures that onset ≤12 months was significantly associated (*p* = 4.127 × 10–7) with more severe intellectual disability, compared with onset >12 months (4). The first year after birth was an important period when the metabolism was increased in the frontal cortex and the brain developed rapidly (11, 12). They speculated that the early onset of seizures within the first year might disrupt neural development and lead to cognitive dysfunction. However, a recent study of genotype and phenotype of *PCDH19* demonstrated that the late-onset seizures accompanied by milder intellectual disability occurred in patients with truncating variants located on extracellular cadherin (EC) domain 5 to the cytoplasmic domain compared with those of patients with other variants (5). The earlier seizures onset time accompanied by more severe cognitive impairment and psychological and behavioral abnormalities likely reflected a manifestation of more severe cellular signal interference. Early onset and severe cellular signal interference may be mutually influenced, resulting in the presence of a severe phenotype.

The proportion of *PCDH19* mosaic variation in males seemed to be associated with seizure severity. There was a striking sorting through adhesion specificity in a combinatorial manner between cells expressing wild-type (WT) *PCDH19* and null *PCDH19* in the developing cortex (13). This might be explained that homophilic trans (cell–cell) interactions were preferred for all δ-protocadherins family (14). Heterozygous mutation of *PCDH19* in mice revealed that a mismatch between *PCDH19* and N-cadherin (Ncad) impairs Ncad-dependent β-catenin signaling and mossy fiber presynaptic development (15). Theoretically, the phenotype tended to be worse when the ratio of *PCDH19* mutation and normal cells was close to 50%. In the previously reported cases, epilepsy and developmental disorders were mild in most cases when the mutation mosaicism rate in peripheral blood was high or low (6, 7). However, it appeared that the mosaic ratio in dermal fibroblasts better reflected the severity of the disorder than in peripheral blood cells. The patient in our study presented a mild to moderate phenotype with mosaic ratios of 27.9% in buccal mucosal cells, 48.3% in exfoliated cells in urine, and 50.6% in peripheral blood of the proband. There have been several asymptomatic male cases with mosaic mutation of *PCDH19* detected from peripheral blood cell (16, 17), in particular, one of them revealed approximately the same ratio (50:50) between the *PCDH19* variant allele and the wild-type allele, but this variant was not identified in skin fibroblasts. Conversely, a male patient showed severe epilepsy with a normal *PCDH19* allele in 53% of the fibroblasts despite no *PCDH19* mutation signal in peripheral blood lymphocytes (7). It seemed that the mosaic proportion of dermal fibroblasts resembled one of the nerve cells because the two originate from the ectoderm of the embryo, which could serve as a marker for predicting the severity and prognosis of *PCDH19* in epilepsy.

The boy in our study was treated at the first onset with prednisone and antiseizure drugs to improve neural function and control seizures, respectively. The low steroid can be associated with severe *PCDH19*-related encephalopathy. Interestingly, there were multiple defects in peripheral steroidogenesis in female patients with *PCDH19* mutations, but restoration of adrenal steroidogenesis was beneficial for the increase in postpubertal *PCDH19* febrile epilepsy (18). Furthermore, corticosteroids could suppress the seizure clusters immediately in *PCDH19* female epilepsy (19). The potential pharmacological mechanism of steroids in *PCDH19* encephalopathy had two facets, (1) The steroid ameliorated the blood-brain barrier (BBB) vulnerability resulting from the *PCDH19* variation (19). (2) As an antiepileptic agent, the neurosteroids could enhance both tonic and phasic γ-aminobutyric acid receptor A (GABAA) dependent inhibitory currents (18). Additionally, in this case we observed the long-term control (>12 months) of epilepsy by anti-epileptic drugs, including Depakin and Keppra. We speculated that there were two reasons, including the late onset time of seizures and the potent efficacy of valproate and levetiracetam, which had antiepileptic response rates of 61 and 57%, respectively, after 12 months of use (20, 21). In short, this study provided evidence for *PCDH19*-related epilepsy therapy and confirmed the utility of steroids and valproate and levetiracetam in controlling seizures caused by the mosaic *PCDH19* mutation.

## Conclusion

This study reported a novel, *de novo*, mosaic and a nonsense mutation (c.840C > A, pTyr280^*^) in a 6 year old boy with febrile epilepsy, expanding the genotypes and broadening the spectrum. This study emphasized that the *PCDH19* mosaic variation should not be overlooked in males with seizures. The whole-exome sequence should be initiated for patients who are characterized by febrile seizures, cognitive impairment, and neuropsychiatric disorders.

## Data availability statement

The datasets presented in this article are not readily available because of ethical and privacy restrictions. Requests to access the datasets should be directed to the corresponding authors.

## Ethics statement

The studies involving human participants were reviewed and approved by the Ethics Committee of Guangzhou Women and Children's Medical Center. Written informed consent to participate in this study was provided by the participants' legal guardian/next of kin. Written informed consent was obtained from the individual(s), and minor(s)' legal guardian/next of kin, for the publication of any potentially identifiable images or data included in this article.

## Author contributions

Design, writing, and editing manuscript: GC, HZ, and JH. Analysis and interpretation of sequence data for the work: HZ, YLu, and YW. Clinical information and follow-up: YLi, JX, KC, and RH. All authors contributed to the article and approved the submitted version.

## Funding

This study was supported by Guangdong Basic and Applied Basic Research Foundation (2021A1515220070) and Guangzhou Women and Children's Medical Center (GWCMC2020-6-007).

## Conflict of interest

The authors declare that the research was conducted in the absence of any commercial or financial relationships that could be construed as a potential conflict of interest.

## Publisher's note

All claims expressed in this article are solely those of the authors and do not necessarily represent those of their affiliated organizations, or those of the publisher, the editors and the reviewers. Any product that may be evaluated in this article, or claim that may be made by its manufacturer, is not guaranteed or endorsed by the publisher.

## References

[1] Symonds JD McTague A. Epilepsy and developmental disorders: next generation sequencing in the clinic. Eur J Paediatr Neurol. (2020) 24:15–23. 10.1016/j.ejpn.2019.12.008 31882278

[2] Dell'Isola GB Vinti V Fattorusso A Tascini G Mencaroni E Di Cara G. The broad clinical spectrum of epilepsies associated with protocadherin 19 gene mutation. Front Neurol. (2021) 12:780053. 10.3389/fneur.2021.780053 35111125 PMC8801579

[3] Samanta D. Pcdh19-related epilepsy syndrome: a comprehensive clinical review. Pediatr Neurol. (2020) 105:3–9. 10.1016/j.pediatrneurol.2019.10.009 32057594

[4] Kolc KL Sadleir LG Scheffer IE Ivancevic A Roberts R Pham DH. A systematic review and meta-analysis of 271 Pcdh19-variant individuals identifies psychiatric comorbidities, and association of seizure onset and disease severity. Mol Psychiatry. (2019) 24:241–51. 10.1038/s41380-018-0066-9 29892053 PMC6344372

[5] Shibata M Ishii A Goto A Hirose S. Comparative characterization of pcdh19 missense and truncating variants in Pcdh19-related epilepsy. J Hum Genet. (2021) 66:569–78. 10.1038/s10038-020-00880-z 33262389 PMC8144015

[6] de Lange IM Rump P Neuteboom RF Augustijn PB Hodges K Kistemaker AI. Male patients affected by mosaic Pcdh19 mutations: five new cases. Neurogenetics. (2017) 18:147–53. 10.1007/s10048-017-0517-5 28669061 PMC5522515

[7] Depienne C Bouteiller D Keren B Cheuret E Poirier K Trouillard O. Sporadic infantile epileptic encephalopathy caused by mutations in Pcdh19 resembles dravet syndrome but mainly affects females. PLoS Genet. (2009) 5:e1000381. 10.1371/journal.pgen.1000381 19214208 PMC2633044

[8] Morishita H Yagi T. Protocadherin family: diversity, structure, and function. Curr Opin Cell Biol. (2007) 19:584–92. 10.1016/j.ceb.2007.09.006 17936607

[9] Dibbens LM Tarpey PS Hynes K Bayly MA Scheffer IE Smith R. X-Linked protocadherin 19 mutations cause female-limited epilepsy and cognitive impairment. Nat Genet. (2008) 40:776–81. 10.1038/ng.149 18469813 PMC2756413

[10] Wolverton T Lalande M. Identification and Characterization of Three Members of a Novel Subclass of Protocadherins. Genomics. (2001) 76:66–72. 10.1006/geno.2001.6592 11549318

[11] Chugani HT. A critical period of brain development: studies of cerebral glucose utilization with pet. Prevent Med. (1998) 27:184–8. 10.1006/pmed.1998.0274 9578992

[12] Knickmeyer RC Gouttard S Kang C Evans D Wilber K Smith JK. A structural Mri study of human brain development from birth to 2 years. J Neurosci. (2008) 28:12176–82. 10.1523/JNEUROSCI.3479-08.2008 19020011 PMC2884385

[13] Pederick DT Richards KL Piltz SG Kumar R Mincheva-Tasheva S Mandelstam SA. Abnormal cell sorting underlies the unique X-linked inheritance of Pcdh19 epilepsy. Neuron. (2018) 97:59–66. 10.1016/j.neuron.2017.12.005 29301106

[14] Harrison OJ Brasch J Katsamba PS Ahlsen G Noble AJ Dan H. Family-wide structural and biophysical analysis of binding interactions among non-clustered delta-protocadherins. Cell Rep. (2020) 30:2655–71. 10.1016/j.celrep.2020.02.003 32101743 PMC7082078

[15] Hoshina N Johnson-Venkatesh EM Hoshina M Umemori H. Female-specific synaptic dysfunction and cognitive impairment in a mouse model of Pcdh19 disorder. Science. (2021) 372:eaaz3893. 10.1126/science.aaz3893 33859005 PMC9873198

[16] Kolc KL Moller RS Sadleir LG Scheffer IE Kumar R Gecz J. Pcdh19 pathogenic variants in males: expanding the phenotypic spectrum. Adv Exp Med Biol. (2020) 1298:177–87. 10.1007/5584_2020_574 32852734

[17] Liu A Yang X Yang X Wu Q Zhang J Sun D. Mosaicism and incomplete penetrance of Pcdh19 mutations. J Med Genet. (2019) 56:81–8. 10.1136/jmedgenet-2017-105235 30287595 PMC6581080

[18] Trivisano M Lucchi C Rustichelli C Terracciano A Cusmai R Ubertini GM. Reduced steroidogenesis in patients with Pcdh19-female limited epilepsy. Epilepsia. (2017) 58:e91–e5. 10.1111/epi.13772 28471529

[19] Higurashi N Takahashi Y Kashimada A Sugawara Y Sakuma H Tomonoh Y. Immediate suppression of seizure clusters by corticosteroids in Pcdh19 female epilepsy. Seizure. (2015) 27:1–5. 10.1016/j.seizure.2015.02.006 25891919

[20] Lotte J Bast T Borusiak P Coppola A Cross JH Dimova P. Effectiveness of antiepileptic therapy in patients with Pcdh19 mutations. Seizure. (2016) 35:106–10. 10.1016/j.seizure.2016.01.006 26820223

[21] Sadleir LG Kolc KL King C Mefford HC Dale RC Gecz J. Levetiracetam efficacy in Pcdh19 girls clustering epilepsy. Eur J Paediatr Neurol. (2020) 24:142–7. 10.1016/j.ejpn.2019.12.020 31928905

